# Angiotensin II Type I Receptor Antagonism Attenuates Nicotine-Induced Cardiac Remodeling, Dysfunction, and Aggravation of Myocardial Ischemia-Reperfusion Injury in Rats

**DOI:** 10.3389/fphar.2019.01493

**Published:** 2019-12-12

**Authors:** Anand Ramalingam, Siti Balkis Budin, Norsyahida Mohd. Fauzi, Rebecca H. Ritchie, Satirah Zainalabidin

**Affiliations:** ^1^Programme of Biomedical Science, Centre for Applied and Health Sciences, Faculty of Health Sciences, Universiti Kebangsaan Malaysia, Kuala Lumpur, Malaysia; ^2^Drug and Herbal Research Centre, Faculty of Pharmacy, Universiti Kebangsaan Malaysia, Kuala Lumpur, Malaysia; ^3^Heart Failure Pharmacology, Baker Heart and Diabetes Institute, Melbourne, VIC, Australia; ^4^Drug Discovery Biology, Monash Institute of Pharmaceutical Sciences, Monash University, Parkville, VIC, Australia

**Keywords:** cardiac fibrosis, hypertension, inflammation, irbesartan, oxidative stress

## Abstract

Increased exposure to nicotine contributes to the development of cardiac dysfunction by promoting oxidative stress, fibrosis, and inflammation. These deleterious events altogether render cardiac myocytes more susceptible to acute cardiac insults such as ischemia-reperfusion (I/R) injury. This study sought to elucidate the role of angiotensin II type I (AT1) receptors in cardiac injury resulting from prolonged nicotine administration in a rat model. Male Sprague-Dawley rats were given nicotine (0.6 mg/kg ip) for 28 days to induce cardiac dysfunction, alone or in combination with the AT1 receptor antagonist, irbesartan (10 mg/kg, po). Vehicle-treated rats were used as controls. Rat hearts isolated from each experimental group at study endpoint were examined for changes in function, histology, gene expression, and susceptibility against acute I/R injury determined *ex vivo*. Rats administered nicotine alone exhibited significantly increased cardiac expression of angiotensin II and angiotensin-converting enzyme (ACE) in addition to elevated systolic blood pressure (SBP) and heart rate. Furthermore, nicotine administration markedly reduced left ventricular (LV) performance with concomitant increases in myocardial oxidative stress, fibrosis, and inflammation. Concomitant treatment with irbesartan attenuated these effects, lowering blood pressure, heart rate, oxidative stress, and expression of fibrotic and inflammatory genes. Importantly, the irbesartan-treated group also manifested reduced susceptibility to I/R injury *ex vivo*. These findings suggest that AT1 receptors play an important role in nicotine-induced cardiac dysfunction, and pharmacological approaches targeting cardiac AT1 receptors may thus benefit patients with sustained exposure to nicotine.

## Introduction

Prolonged exposure to nicotine *via* cigarette smoking, chewable tobacco, as well as nicotine inhalation devices is associated with an increased risk of cardiovascular diseases (McEvoy et al., 2015; Kim et al., 2017; Kunutsor et al., 2018). In animal models, prolonged administration of nicotine not only promotes vascular endothelial dysfunction associated with hypertension, but also directly impacts the cardiac structure and function, promoting oxidative stress, inflammation, fibrosis, and cardiomyocyte apoptosis (Joukar et al., 2012; Zainalabidin et al., 2014; Ramalingam et al., 2016; Si et al., 2017a; Li et al., 2018). In several lines of evidence, nicotine was shown to induce cardiac damage independent of hypertension, directly accounting for significant reduction in cardiac function (Hu et al., 2011). In addition, nicotine can also aggravate myocardial ischemia-reperfusion (I/R) injury. Administration of nicotine, either on acute exposure or with chronic administration prior to surgical induction of I/R injury, robustly exaggerated myocyte loss and functional impairment, as well as oxidative stress in both canine and rat models in a dose-dependent fashion (Przyklenk, 1994; Schrör et al., 1998).

Oxidative stress has been broadly implicated as a major cause of nicotine-induced cardiovascular abnormalities both *in vivo* and *in vitro*. Indeed, supplementation with exogenous antioxidants such as vitamin E orally as well as through metallothionein overexpression; effectively prevented nicotine-induced cardiac damage in these animal models (Demiralay et al., 2007; Erat et al., 2007; Gumustekin et al., 2010; Hu et al., 2011). Although it is suggested that nicotine-induced upregulation of reactive oxygen species (ROS) production drives cardiac hypertrophy, fibrosis, and inflammation upon chronic exposure (Hu et al., 2011; Ramalingam et al., 2016), limited data is available to describe precise mechanisms underlying nicotine-induced oxidative stress and cardiac dysfunction *in vivo*. Renin angiotensin system (RAS) is a known regulator of ROS and oxidative stress in other models of cardiovascular diseases such as hypertension, diabetic cardiomyopathy, and heart failure (Huynh et al., 2013; Nagatomo et al., 2014; Wysocki et al., 2014; Wang et al., 2018). Despite evidence for the involvement of oxidative stress in nicotine-induced cardiac pathophysiology, the role of angiotensin II type I (AT1) receptors known to regulate cardiovascular ROS levels in other context, however have not been elucidated in settings of prolonged nicotine administration. Such a role is particularly relevant given that nicotine increases expression of angiotensin-converting enzymes (ACE and ACE2) (Ljungberg and Persson, 2008).

This study therefore sought to i) determine the impact of prolonged nicotine administration on expression of cardiac angiotensin II (ANG II) and its receptor system, as well as ii) investigate whether targeting AT1 receptors using a conventional antagonist, irbesartan prevents nicotine-induced cardiac remodeling, dysfunction, and the associated aggravation of myocardial I/R injury in rat model.

## Materials and Methods

### Animals

Male Sprague-Dawley rats (5–6 weeks old, 180–230 g) were obtained from Synertec Enterprise (Kuala Lumpur, Malaysia) and were housed under standard laboratory conditions in UKM Kuala Lumpur Campus Animal Facility for acclimatization prior to any experiments. Standard rodent pellet and tap water were provided *ad libitum*. Throughout acclimatization, each rat was inspected carefully for food and water intake, weight gain, signs of distress, and injuries. Animals showing reduced weight gain, hunching, or bleeding injuries were all excluded from subsequent experiments. All procedures involving animals in this study adhered to the ethical guidelines provided by the UKM Animal Ethics Committee (UKMAEC) under the project code of FSK/BIOMED/2012/SATIRAH/12-DEC./486-DEC.-2012-DEC.2014.

### Study Design

After acclimatization, all rats were randomly allotted into three experimental groups, namely 1) vehicle control (n=13), 2) nicotine alone (NIC) (n = 14), and 3) nicotine plus irbesartan (NIC+Irb) (n = 14). Rats from both NIC and NIC+Irb groups were given 0.6 mg/kg nicotine dissolved in normal saline *via* intraperitoneal (ip) injection for 28 days as previously described (Ramalingam et al., 2016). These rats also received either dimethylsulfoxide (DMSO) vehicle alone or irbesartan (10 mg/kg in DMSO) *via* oral feeding respectively (Watanabe et al., 2015). Vehicle control rats received DMSO vehicle alone for the duration of this study. DMSO vehicle (5% v/v) was prepared using sterile distilled water. Throughout this study, *in vivo* measurements of systolic blood pressure (SBP) and heart rate were obtained at weekly intervals *via* the non-invasive tail cuff method (CODA^™^ non-invasive blood pressure system, Kent Scientific, USA) as previously described (Si et al., 2017b). All rats were habituated to the CODA^™^ system in a designated quiet room (27 ± 2°C) for at least three consecutive days prior to acquisition of baseline measurements.

At study end, blood was collected from each animal *via* orbital sinus bleeding, for assessment of plasma cotinine using ELISA kit from Elabscience Biotechnology (Wuhan, China). Hearts collected from a subset of rats (n = 6–7 per group; n = 6 for control, n = 6 for NIC and n = 7 for NIC+Irb) were used for analysis of heart structure (histology and immunohistochemistry), gene expression, mitochondrial ROS production, and antioxidant activity. Another subset of rats (n = 7–8/group; n = 7 for control, n = 8 for NIC, and n = 7 for NIC+Irb) were used for Langendorff heart preparation to study changes in cardiac function and myocardial susceptibility to I/R injury.

### Histology and Immunohistochemistry

Left ventricular (LV) tissues were fixed in neutral-buffered formalin, processed in a graded series of alcohol (50, 70, 85, 90, 95, and 100%), cleared in two changes of xylene and embedded in paraffin. LV tissue sections (5 µm) were then stained with hematoxylin and eosin (H&E) for measurement of cardiomyocyte size or picrosirius red for measurement of collagen density in bright field microscopic images (Ali et al., 2018). For H&E staining, cross-sectional area (CSA) of ∼100 cardiomyocytes per animal were quantified using ImageJ software from 10 independent bright-field images acquired under 40X magnification. Collagen density was also quantified using ImageJ software with macro from 10 independent bright-field images acquired under 10X magnification (Huynh et al., 2013).

LV sections (5 µm) were also used for immune-detection of oxidative stress marker, 3-nitrotyrosine (Huynh et al., 2013). Tissues were subjected to peroxidase quenching using 3% hydrogen peroxide in methanol and blocking with 5% normal horse serum in Tris-buffered saline (TBS) for 1 h at room temperature. LV sections were then incubated with monoclonal antibodies against 3-nitrotyrosine (1:250, Santa Cruz Biotechnology, USA) overnight at 4°C prior to biotinylated anti-mouse secondary antibodies for 30 min (1:250, Vector Laboratories, USA) and avidin-biotin complex reagent for 30 min at room temperature (VECTASTAIN Elite ABC Kit, Vector Laboratories, USA). Sections were developed with 3,3’-diaminobenzidine reaction mixture (DAB Peroxidase HRP Substrate Kit, Vector Laboratories, USA) and were mounted in dibutylphthalate polystyrene xylene mounting medium. Bright field microscopic images were acquired under 40X magnification using Olympus microscope and were analyzed using ImageJ software (NIH, USA).

### Analysis of Gene Expression

Total RNA from LV tissues was extracted using QIAzol lysis reagent (QIAGEN, Germany) and was reverse transcribed as previously described (Ali et al., 2018). Briefly, 2 µg purified RNA was reverse transcribed in a 20-µl reaction mixture containing reverse transcription enzyme, deoxyribonucleotide triphosphates, Mg^2+^, and reaction buffer (QuantiNova^™^ Reverse Transcription Kit, QIAGEN, Germany). Expression of pro-hypertrophic genes (atrial natriuretic peptide, ANP; brain natriuretic peptide, BNP), fibrotic genes (transforming growth factor β1, TGFβ1; fibronectin, FN1), inflammation-related genes (tumor necrosis factor α, TNFα; interleukin 6, IL6; interleukin 10, IL10; annexin A1, ANXA1; formyl peptide receptor 2, FPR2), oxidative stress gene (NOX2 subunit of NADPH oxidase and superoxide dismutase, SOD2), angiotensin converting enzymes (ACE, ACE2), as well as ANG II receptors (AT1 and AT2) were determined using the QuantiNova^™ ^SYBR Green PCR Kit (QIAGEN, Germany). Quantitative analysis of gene expression was performed using Applied Biosystems Prism^®^ 7700 Sequence Detection Software, using the primer sequences generated from the GenBank^®^ (**Table 1**). Comparative 2^-ΔΔCt^ method to used detect fold differences relative to the vehicle control group with ribosomal 18S as the housekeeping gene (De Blasio et al., 2017). If cycle threshold (Ct) for the gene of interest exceed 40 (i.e., undetermined result), expression was reported as zero or not detected.

**Table 1:** Primer sequence for quantitative real time PCR analysis.

Gene name	Primer sequence (5’ to 3’)	
18s	Forward	TTCGAGGCCCTGTAATTGGA
	Reverse	GCAGCAACTTTAATATAGGCTATTGG
Ace	Forward	ATCCTGGCTTCCTCACGAAA
	Reverse	CTCCTGTGTCTGAGAAGCCA
Ace2	Forward	TTCCCAGAGAACAGTGGACC
	Reverse	TGTGTAGTGGGCCATCATGT
At1	Forward	CAAAAGGAGATGGGAGGTCA
	Reverse	TGACAAGCAGTTTGGCTTTG
At2	Forward	CAACTGGCACCAATGAGTCC
	Reverse	GGAAGGGTTGCCAAAAGGAG
Anp	Forward	GGAAGTCAACCCGTCTCAGA
	Reverse	TGGGCTCCAATCCTGTCAAT
Bnp	Forward	ACAAGAGAGAGCAGGACACC
	Reverse	TCTGGAGACTGGCTAGGACT
Tgfb1	Forward	CCTGCAAGACCATCGACATG
	Reverse	TGTTGTACAAAGCGAGCACC
Fn1	Forward	GAAAGGCAACCAGCAGAGTC
	Reverse	CTGGAGTCAAGCCAGACACA
Nox2	Forward	CTGCCAGTGTGTCGGAATCT
	Reverse	TGTGAATGGCCGTGTGAAGT
Tnfa	Forward	ACACACGAGACGCTGAAGTA
	Reverse	GGAACAGTCTGGGAAGCTCT
Il6	Forward	TCTCTCCGCAAGAGACTTCCA
	Reverse	ATACTGGTCTGTTGTGGGTGG
Il10	Forward	CCTGCTCTTACTGGCTGGAG
	Reverse	TGTCCAGCTGGTCCTTCTTT
Anxa1	Forward	GCCCCTACCCTTCCTTCAAT
	Reverse	GCCAAAACAACCTCCTCCAG
Fpr2	Forward	ATCTGGGTAGCTGGATTCCG
	Reverse	GGATGCAGGACACAAATGCA

### Analysis of Mitochondrial Reactive Oxygen Species and Endogenous Antioxidants

Rat heart mitochondria were isolated from LV tissues *via* differential centrifugation in ice-cold sucrose buffer (containing 250 mM sucrose, 20 mM Tris-HCl, 40 mM KCl, and 1 mM ethylenediaminetetraacetic acid, pH 7.5) as previously described (Iglesias-González et al., 2013). Two hundred micrograms of isolated mitochondria were then loaded with 5 µM membrane-permeable fluorescent probe, MitoSOX^™^ Red (Invitrogen, USA) in the dark for 15 min at 37°C (modified from Tran et al., 2012). Fluorescence intensity was measured using Varioskan^™^ multimode plate reader (Thermo Scientific, USA) at 510 nm excitation and 580 nm emission wavelengths and were expressed relative to the vehicle control group. A portion of LV tissues was also homogenized in ice-cold 10 mM Tris-HCl buffer and the supernatant was used for detection of SOD2 activity as well as glutathione-to-glutathione disulphide ratio (GSH : GSSG) as previously described (Beyer and Fridovich, 1987; Rahman et al., 2006). Results were expressed as per mg protein content in the tissue homogenate that was pre-determined using Bradford protein assay (modified from Bradford, 1976).

### Langendorff Perfusion and Ischemia-Reperfusion Injury *Ex Vivo*

For Langendorff heart studies, rats from each group were given sodium heparin (500 U/kg) as anticoagulant and urethane (1.2 g/kg) for anesthesia *via* intraperitoneal injections (Lim et al., 2016). Hearts were rapidly excised and subjected to retrograde perfusion *via* the aortic cannula under constant pressure of ∼60–70 mmHg, with Krebs-Henseleit buffer (containing 118 mM NaCl, 25 mM NaHCO_3_, 4.7 mM KCl, 1.2 mM KH_2_PO_4_, 1.2 mM MgSO_4_, 1.25 mM CaCl_2_, 11 mM glucose; 95% O_2_/5% CO_2_ maintained at 37°C). Changes in coronary flow (CF) and perfusion pressure were monitored continuously using flow and pressure transducers. A water-filled latex balloon was inserted into the LV to monitor changes in heart rate and intraventricular pressure derivatives such as left ventricular systolic pressure (LVSP), left ventricular end-diastolic pressure (LVEDP), maximum velocity of contraction (LV+dP/dt), as well as maximum velocity of relaxation (LV-dP/dt). Changes in left ventricular developed pressure (LVDP) were calculated as the difference between LVEDP and LVSP. All data were acquired using LabChart Pro version 7.0 acquisition software (AD Instruments, Australia). Latex balloon was filled with ∼150 µl water for all rats and the balloon was adjusted carefully to achieve a baseline LVEDP of 0–5 mmHg.

Rat hearts were equilibrated for 20 min with steady-flow and hearts exhibiting poor function (e.g., heart rate <100 beats/min and LV+dP/dt < 1,500 mmHg/sec) during equilibration were excluded from the study (Chin et al., 2016). After equilibration, each heart was subjected to 20 min of global ischemia and 60 min of reperfusion (Whittington et al., 2013). The heart was immersed in water-jacketed glass chamber throughout the procedure to maintain heart temperature at 37°C. Coronary effluent was collected at 10-min intervals throughout reperfusion, for measurement of cardiac injury markers: cardiac troponin T (cTnT) and lactate dehydrogenase (LDH) using ELISA kits (Elabscience Biotechnology, Wuhan, China) and spectrophotometric assay (Wroblewski and Ladue, 1955).

### Statistical Analysis

All data are presented as mean ± standard error of mean (SEM). One-way or two-way analysis of variance (ANOVA) followed by a Tukey’s *post-hoc* test was used to analyze differences between groups, unless otherwise mentioned. Statistical significance was considered at *P* < 0.05.

## Results

### Systemic Characteristics

**Figure 1:**
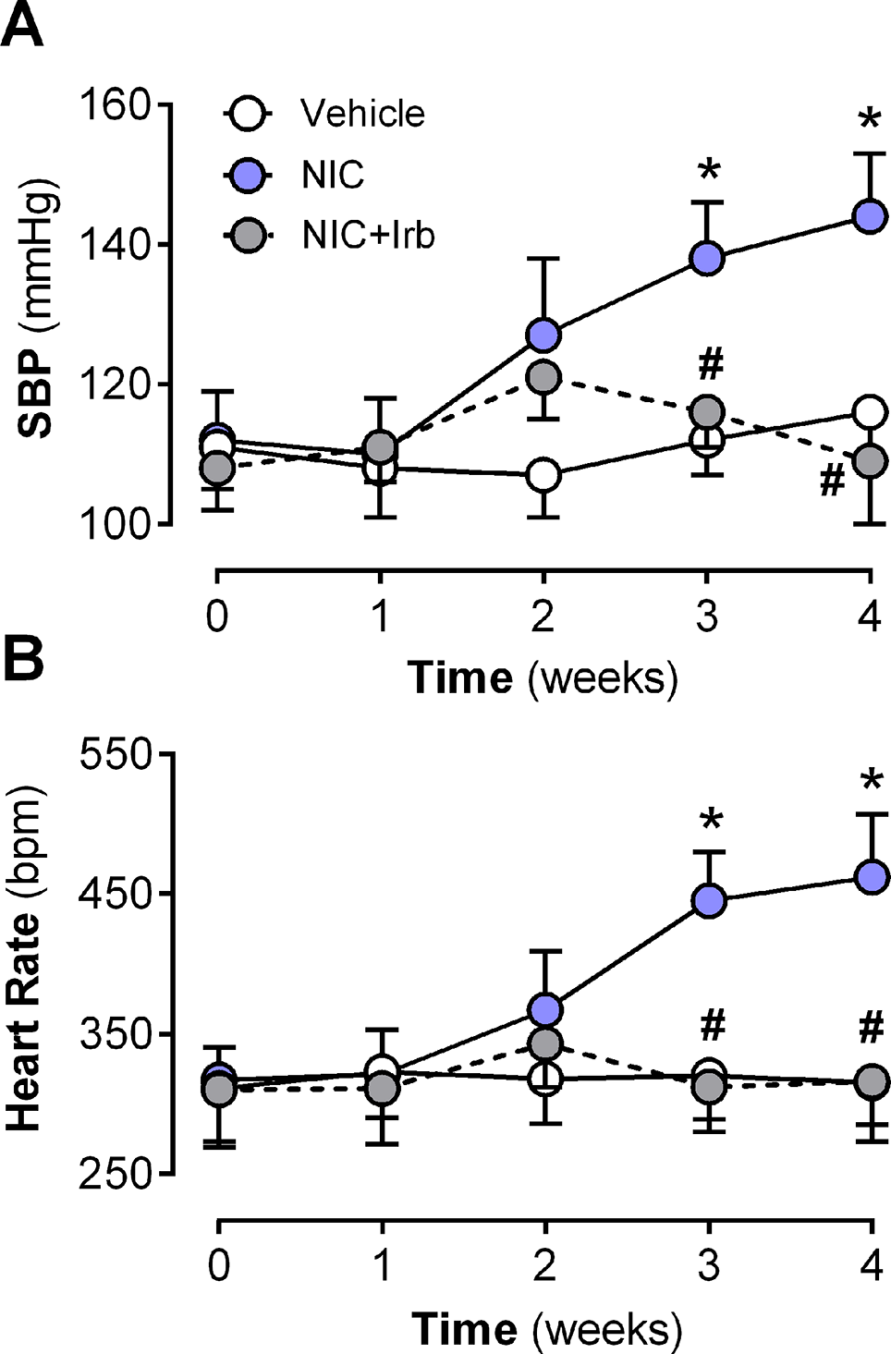
Time-course for changes in **(A)** systolic blood pressure, systolic blood pressure, and **(B)** heart rate in rats administered with nicotine and irbesartan for 4 weeks. All values are given as mean ± SEM for n = 13–14/group; *P < 0.05 for vehicle *vs.* nicotine (NIC) group; and ^#^ *P* < 0.05 for NIC *vs.* NIC+Irb group using two-way ANOVA with Tukey *post-hoc* test.

Neither nicotine nor irbesartan administration significantly altered body weight of rats after 4 weeks of administration (**Table 2**). End-point analysis of organ weights however revealed significantly increased heart weight, LV weight, heart weight-to-tibia length ratio (HW : TL), and left ventricle weight-to-tibia length ratio (LV: TL) in nicotine-administered rats (all *P* < 0.05 *vs.* vehicle control; **Table 2**). Irbesartan exhibited a tendency to attenuate these changes, compared to nicotine-administered rats, but failed to attain significance (all *P* < 0.1; **Table 2**). Cotinine levels measured in plasma as a biomarker for nicotine intake was comparable in the NIC and NIC+Irb groups (**Table 2**). Nicotine was also shown to induce increases in SBP and heart rate compared to vehicle controls across 4 weeks of administration (both *P* < 0.05; **Figure 1**). Both effects were however completely prevented by irbesartan co-administration (both *P* < 0.05; **Figure 1**).

**Table 2:** End-point analysis of body weight, organ weights, and plasma cotinine.

Parameters	Vehicle (n = 13)	NIC (n = 14)	NIC+Irb (n = 14)
Body weight (g)	296 ± 17	280 ± 15	294 ± 121
Heart weight (mg)	1,001 ± 28	1,267 ± 63*	1,073 ± 61†
Atria weight (mg)	34 ± 7	29 ± 5	36 ± 6
LV weight (mg)	510 ± 39	686 ± 50*	523 ± 54†
RV weight (mg)	188 ± 15	181 ± 18	190 ± 16
Lung weight (mg)	1,188 ± 93	1,467 ± 86	1,297 ± 110
Tibia length (mm)	44 ± 3	43 ± 2	42 ± 3
HW : TL (mg/mm)	22.7 ± 0.6	29.5 ± 1.5*	25.6 ± 1.4†
LV : TL (mg/mm)	11.9 ± 1.4	16.1 ± 1.1*	12.3 ± 0.6†
LW : TL (mg/mm)	27.0 ± 2.1	34.1 ± 2.0	30.9 ± 2.6
Cotinine (ng/ml)	–	169 ± 21	142 ± 24

All values are given as mean ± SEM for n = 14-15/group; *p < 0.05 for control vs. NIC group; and ^†^p < 0.1 for NIC vs. NIC+Irb group using one way ANOVA with Tukey post-hoc test. HW, heart weight; LV, left ventricle; LW, lung weight; RV, right ventricle; TL, tibia length.

### Impact of Nicotine on Systemic and Cardiac Angiotensin System

**Figure 2:**
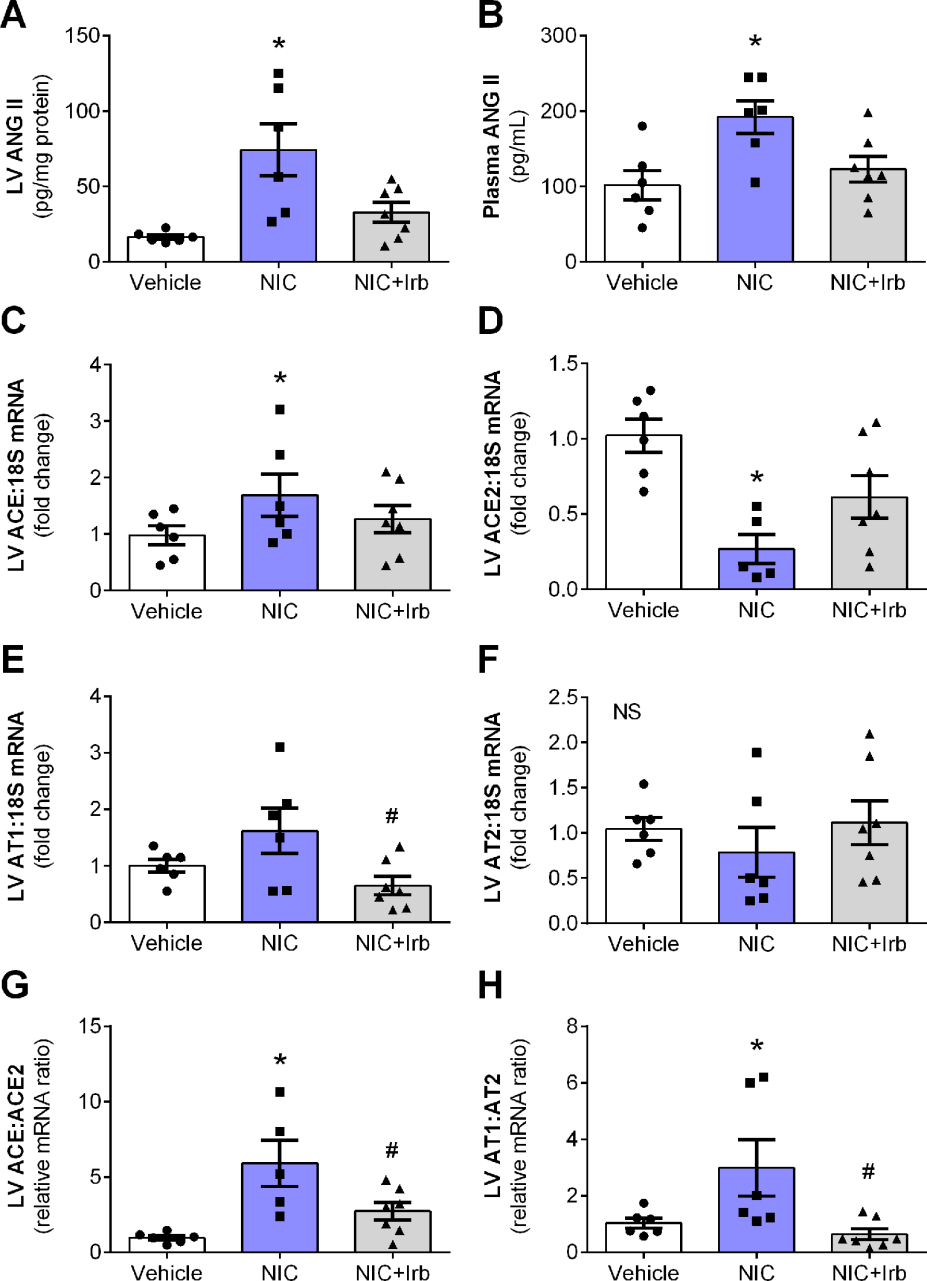
Nicotine dysregulates systemic and cardiac angiotensin system. Levels of ANG II in the **(A)** blood plasma and **(B)** left ventricular (LV) tissues; relative gene expression of **(C)** ACE, **(D)** ACE2, **(E)** AT1, **(F)** AT2 normalized to 18S housekeeping gene, as well as **(G)** ratio of ACE : ACE2 and **(H)** ratio of AT1:AT2 in LV after 4 weeks of nicotine administration in rats. All values are given as mean ± SEM for n = 6 –7/group; *P < 0.05 *vs.* vehicle and ^#^ *P* < 0.05 *vs.* nicotine group using one-way ANOVA with Tukey *post-hoc* test. NS, no significant difference.

After 28 days of administration, nicotine significantly increased circulating and cardiac levels of ANG II (both *P* < 0.05 *vs.* vehicle control; **Figures 2A, B**). This was accompanied by increased LV gene expression of ACE (*P* < 0.05 *vs.* vehicle control; **Figure 2C**), and reduced LV ACE2 expression (*P* < 0.05 *vs.* vehicle control; **Figure 2D**). LV gene expression of both AT1 and AT2 receptors was not significantly affected by nicotine administration (**Figures 2E, F**), however, the ratio of ACE-to-ACE2 as well as AT1-to-AT2 gene expression were both significantly elevated in the nicotine-administered rats compared to vehicle controls (*P* < 0.05; **Figures 2G, H**). Irbesartan significantly attenuated the nicotine-induced increases in gene expression of both ACE-to-ACE2 and AT1-to-AT2 ratios in these rats (all *P* < 0.05; **Figure 2**). Although a tendency for irbesartan to reduce ACE gene expression compared to nicotine alone rats was observed (*P* = 0.1; **Figure 2C**), the AT1 antagonist exhibited no effect on systemic or cardiac ANG II levels and ACE2 as well as AT2 expression (**Figure 2**).

### Impact of AT1 Antagonism on Nicotine-Induced Cardiac Remodeling

**Figure 3:**
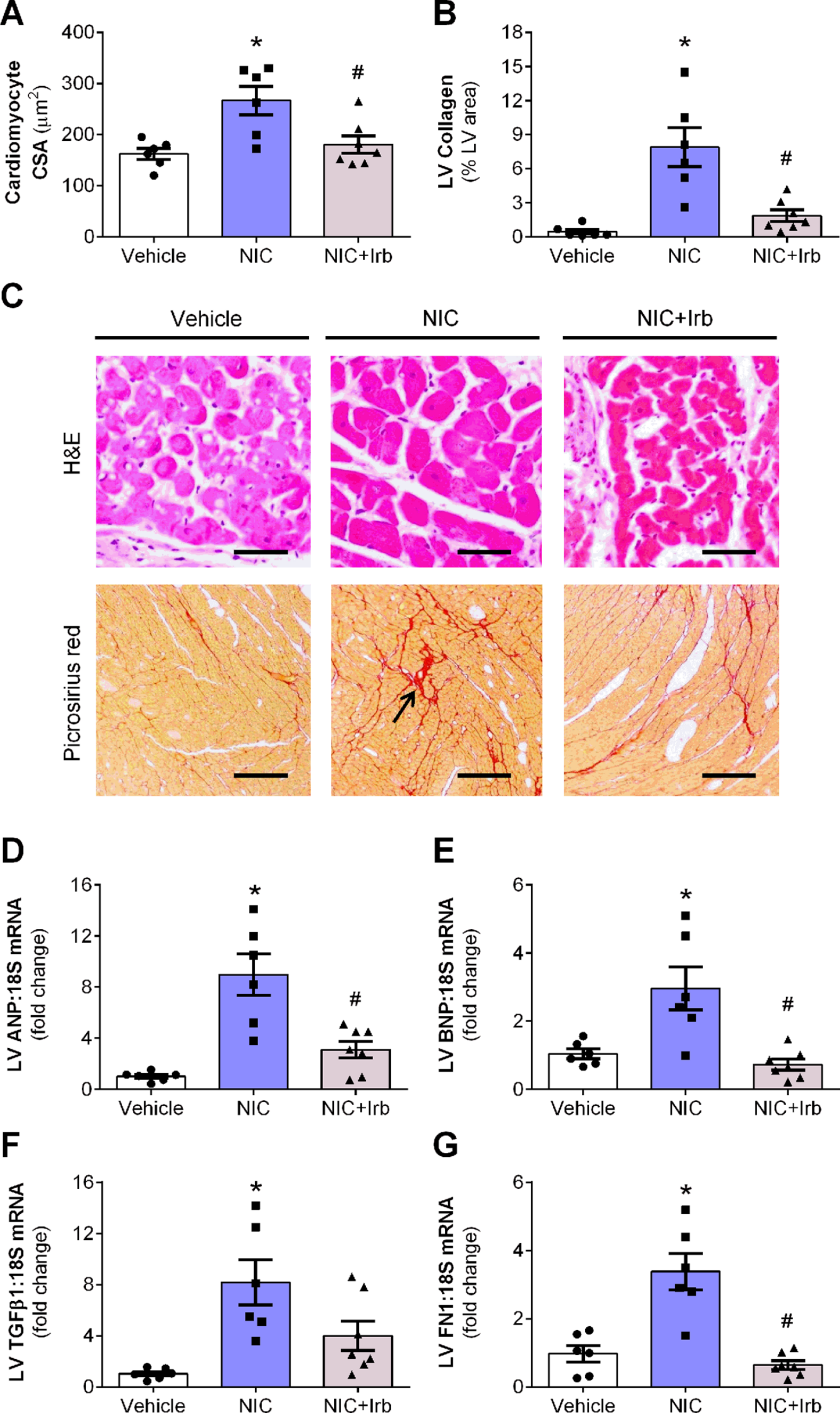
AT1 receptor antagonism attenuates nicotine-induced cardiomyocyte hypertrophy and fibrosis. Quantification of **(A)** cardiomyocyte cross-sectional area, cross-sectional area, and **(B)** collagen density in the left ventricular (LV) tissues with representative images of LV tissues stained with H&E or picrosirius red staining shown in **(C)**. Relative gene expression of **(D)** atrial natriuretic peptide, **(E)** brain natriuretic peptide, **(F)** transforming growth factor β1, and **(G)** FN1 in LV tissues, normalized to 18S housekeeping gene after 4 weeks of nicotine administration. All values are given as mean ± SEM for n = 6 –7/group; *P < 0.05 *vs.* vehicle and ^#^ *P* < 0.05 *vs.* nicotine group using one-way ANOVA with Tukey *post-hoc* test.

Prolonged nicotine administration induced a significant degree of cardiac hypertrophy and fibrosis in rats after 28 days of administration. Computer-assisted analysis of H&E-stained LV tissue sections revealed a marked increase in cardiomyocyte CSA in nicotine-administered rats, compared to vehicle controls (*P* < 0.05; **Figure 3A**). LV collagen density determined using picrosirius red was similarly increased in nicotine-administered rats, compared to vehicle controls (*P* < 0.05; **Figure 3B**). Representative images of LV tissue sections stained with H&E and picrosirius red are shown in **Figure 3C**. Consistent with the increased cardiomyocyte CSA, LV gene expression of ANP and BNP (markers of hypertrophy) were both markedly upregulated in nicotine-administered rats (both *P* < 0.05 *vs.* vehicle controls; **Figures 3D, E**). Nicotine also increased LV gene expression of pro-fibrotic molecules, TGFβ1, and FN1 (both *P* < 0.05 *vs.* vehicle controls; **Figures 3F, G**). Irbesartan significantly blunted nicotine-induced increases in cardiomyocyte CSA, LV collagen, as well as LV expression of ANP, BNP, and FN1 (all *P* < 0.05; **Figure 3**). Irbesartan also tended to lower LV expression of TGFβ1 compared to nicotine alone but there was no significance (*P* = 0.11; **Figure 3F**). Neither nicotine nor irbesartan administration had significant effect on the perivascular fibrosis in rat hearts (**Supplementary File**).

### Impact of AT1 Antagonism on Nicotine-Induced Oxidative Stress

**Figure 4:**
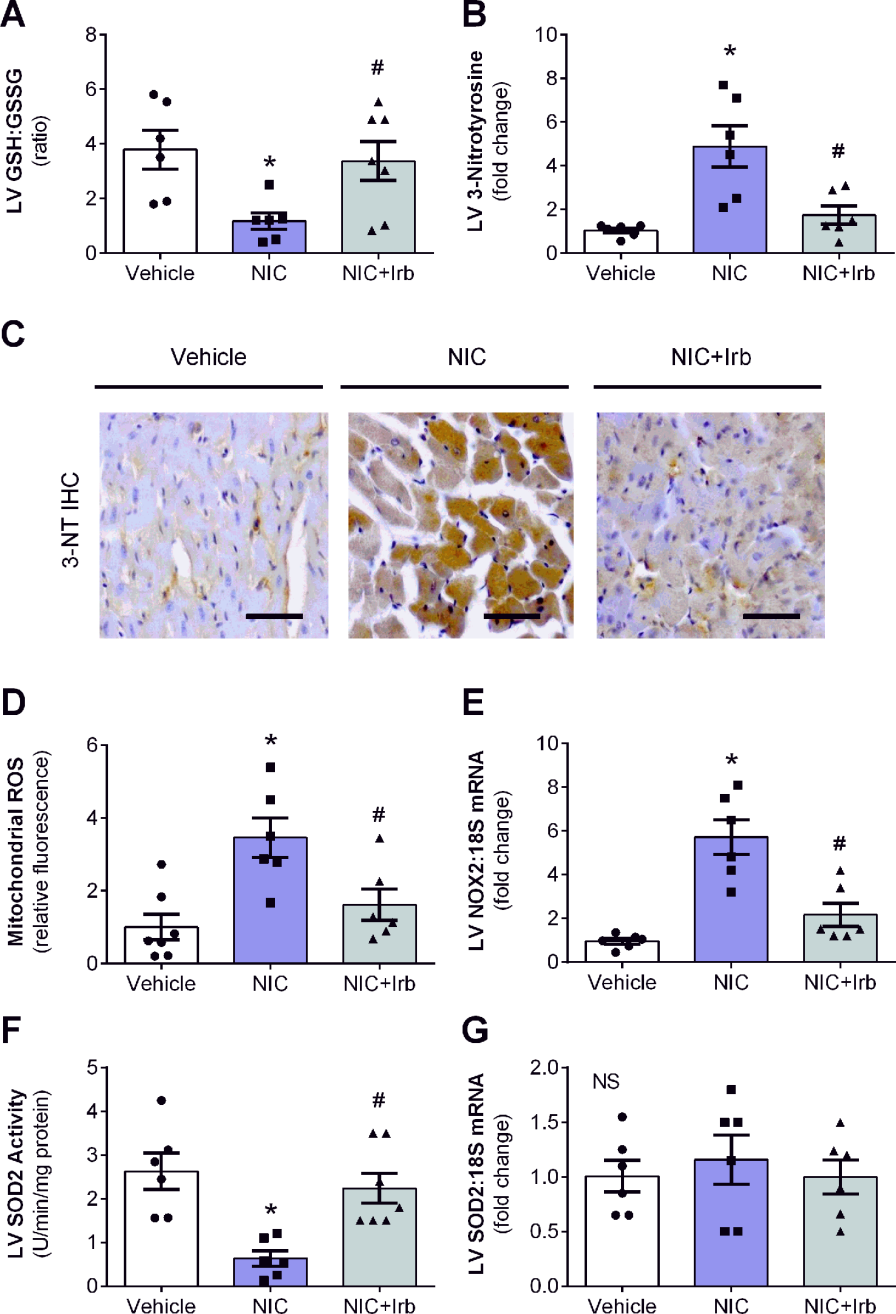
AT1 receptor antagonism blunts nicotine-induced oxidative stress in heart. Quantification of **(A)** glutathione-to-glutathione disulphide ratio as well as **(B)** 3-nitrotyrosine deposition in left ventricular (LV) tissues with representative immunohistochemistry staining images of LV tissues probed for 3-nitrotyrosine (3-NT) shown in **(C)**. **(D)** Mitochondrial reactive oxygen species production determined using MitoSOX in isolated rat heart mitochondria. **(E)** Relative gene expression of NOX2 in LV tissues, normalized to 18S housekeeping gene. **(F)** SOD2 activity and **(G)** Relative gene expression of SOD2 in LV tissues, normalized to 18S housekeeping gene after 4 weeks of nicotine administration. All values are given as mean ± SEM for n = 6 –7/group; *P < 0.05 *vs.* vehicle and ^#^ *P* < 0.05 *vs.* nicotine group using one-way ANOVA with Tukey *post-hoc* test. NS, no significant difference.

LV ratio of GSH : GSSG and levels of 3-nitrotyrosine are both indicators of oxidative stress. In this study, nicotine significantly reduced LV GSH : GSSG after 28 days of administration compared to vehicle controls (*P* < 0.05; **Figure 4A**). Nicotine also increased LV 3-nitrotyrosine content which was detected using immunohistochemistry by ∼4.6-fold relative to the vehicle controls (*P* < 0.05; **Figure 4B**). Irbesartan co-administration effectively attenuated both these markers indicative of reduction in nicotine-induced oxidative stress. Representative images of LV tissue sections immune-stained with antibodies against 3-nitrotyrosine are shown in **Figure 4C**. Apart from these markers, nicotine also significantly up-regulated mitochondrial ROS production and LV gene expression of NOX2 compared to the vehicle controls after 28 days (both *P* < 0.05; **Figures 4D, E**). SOD2 activity in LV tissues collected from nicotine-administered rats was also markedly reduced compared to the vehicle controls, indicative of suppressed antioxidant status in these rats (*P* < 0.05; **Figure 4F**). Conversely, irbesartan effectively blunted nicotine-induced increase in mitochondrial ROS production as well as NOX2 gene expression while concomitantly showing a tendency to preserve SOD2 activity among rats in the NIC+Irb group (**Figures 4D, F**). LV SOD2 gene expression was unaffected by both nicotine and irbesartan despite observed changes in SOD2 activity (**Figure 4G**).

### Impact of AT1 Antagonism on Nicotine-Induced Cardiac Inflammation

**Figure 5:**
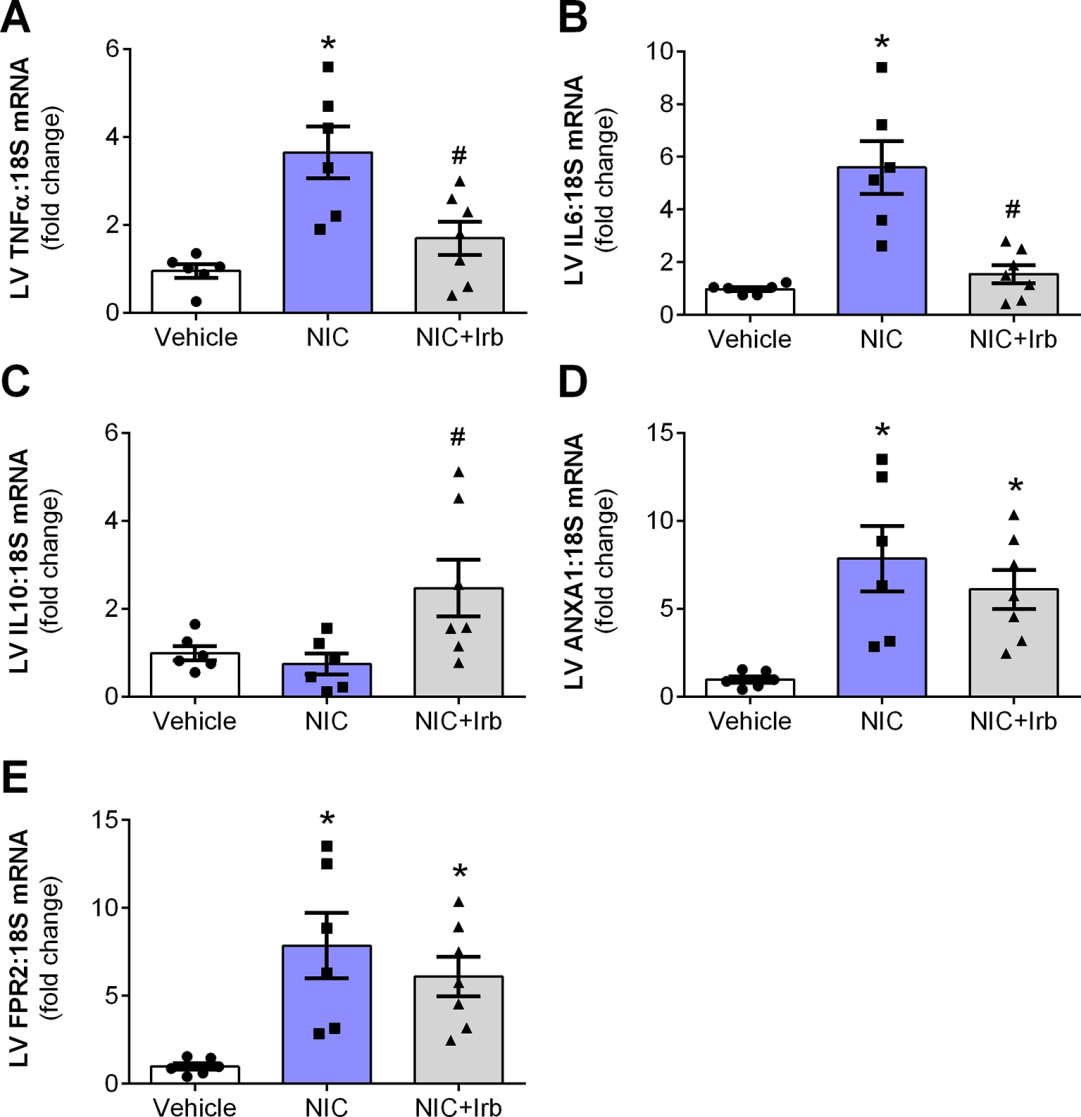
AT1 receptor antagonism limits nicotine-induced cardiac inflammation. Relative gene expression of **(A)** TNFα, **(B)** IL6, **(C)** IL10, **(D)** ANX-A1, and **(E)** FPR2 in left ventricular tissues, normalized to 18S housekeeping gene after 4 weeks of nicotine administration. All values are given as mean ± SEM for n = 6 –7/group; *P < 0.05 *vs.* vehicle and ^#^ *P* < 0.05 *vs.* nicotine group using one-way ANOVA with Tukey *post-hoc* test.

Compared to the vehicle controls, LV gene expression of pro-inflammatory cytokines, TNFα, and IL6 were both significantly elevated in nicotine-administered rats (both *P* < 0.05; **Figures 5A, B**). Irbesartan however, significantly blunted the nicotine-induced increase in LV expression of both TNFα and IL6 (*P* < 0.05). LV gene expression of IL10 was unaffected by 28 days of nicotine administration, irbesartan nevertheless significantly increased expression of this anti-inflammatory cytokine compared to the nicotine-administered rats (*P* < 0.05; **Figure 5C**). Nicotine also significantly increased LV expression of the glucocorticoid-regulated anti-inflammatory protein, ANXA1, and its receptor (FPR2) after 28 days of administration compared to vehicle control rats (both *P* < 0.05; **Figures 5D, E**). Although both ANXA1 and FPR2 remained elevated with concomitant administration of irbesartan, these molecules exhibited a tendency to be blunted compared to nicotine-alone rats (both *P* < 0.1; **Figures 5D, E**).

### Impact of AT1 Antagonism on Nicotine-Induced Cardiac Dysfunction

On Langendorff analysis, we observed that hearts isolated from nicotine-administered rats exhibited poorer LV function compared to control animals at baseline. Markers of LV function (LVSP, LVDP, LV+dP/dt, LV-dP/dt) were all significantly reduced in nicotine-administered rats (all *P* < 0.05 *vs.* vehicle controls; **Table 3**). Irbesartan significantly attenuated this nicotine-induced reduction in LVSP, LVDP and LV-dP/dt (all *P* < 0.05; **Table 3**) and tended to improve LV+dP/dt (*P* = 0.08; **Table 3**). Prolonged nicotine administration markedly impaired baseline CF rate in Langendorff-perfused rat hearts *ex vivo*, which was also prevented by irbesartan (*P* < 0.05; **Table 3**). No significant differences were noted for LVEDP and heart rate at baseline in Langendorff-perfused hearts between all experimental groups (**Table 3**).

**Table 3:** Baseline values of Langendorff-perfused rat hearts from control, nicotine, and NIC+Irb groups, prior to induction of ischemia-reperfusion injury.

Parameters	Vehicle (n = 7)	NIC (n = 8)	NIC+Irb (n = 7)
LVSP (mmHg)	77.6 ± 4.5	50.8 ± 6.6*	69.0 ± 5.9#
LVEDP (mmHg)	2.6 ± 0.5	2.8 ± 0.3	3.1 ± 0.5
LVDP (mmHg)	75.0 ± 4.2	42.0 ± 8.6*	64.5 ± 4.2#
LV +dP/dt (mmHg)	2,480 ± 148	1,743 ± 151*	2,236 ± 180†
LV -dP/dt (mmHg)	1,606 ± 116	1,108 ± 77*	1,645 ± 89#
Coronary flow (ml/min)	11.6 ± 0.6	7.4 ± 0.8*	11.8 ± 0.4#
Heart rate (bpm)	274 ± 36	218 ± 42	262 ± 41

All values are given as mean ± SEM for n = 7 –8/group; *p < 0.05 for control vs. NIC group; and ^#^p < 0.05 for NIC vs. NIC+Irb group; †p < 0.1 for NIC vs. NIC+Irb group using one-way ANOVA with Tukey post-hoc test. LVDP, left ventricular developed pressure; LVEDP, left ventricular end-diastolic pressure; LV+dP/dt, maximal rate of left ventricular contraction; LV –dP/dt, maximal rate of left ventricular relaxation; LVSP, left ventricular systolic pressure.

### Impact of AT1 Antagonism on Nicotine-Induced Post-Ischemic Left Ventricular Dysfunction

**Figure 6:**
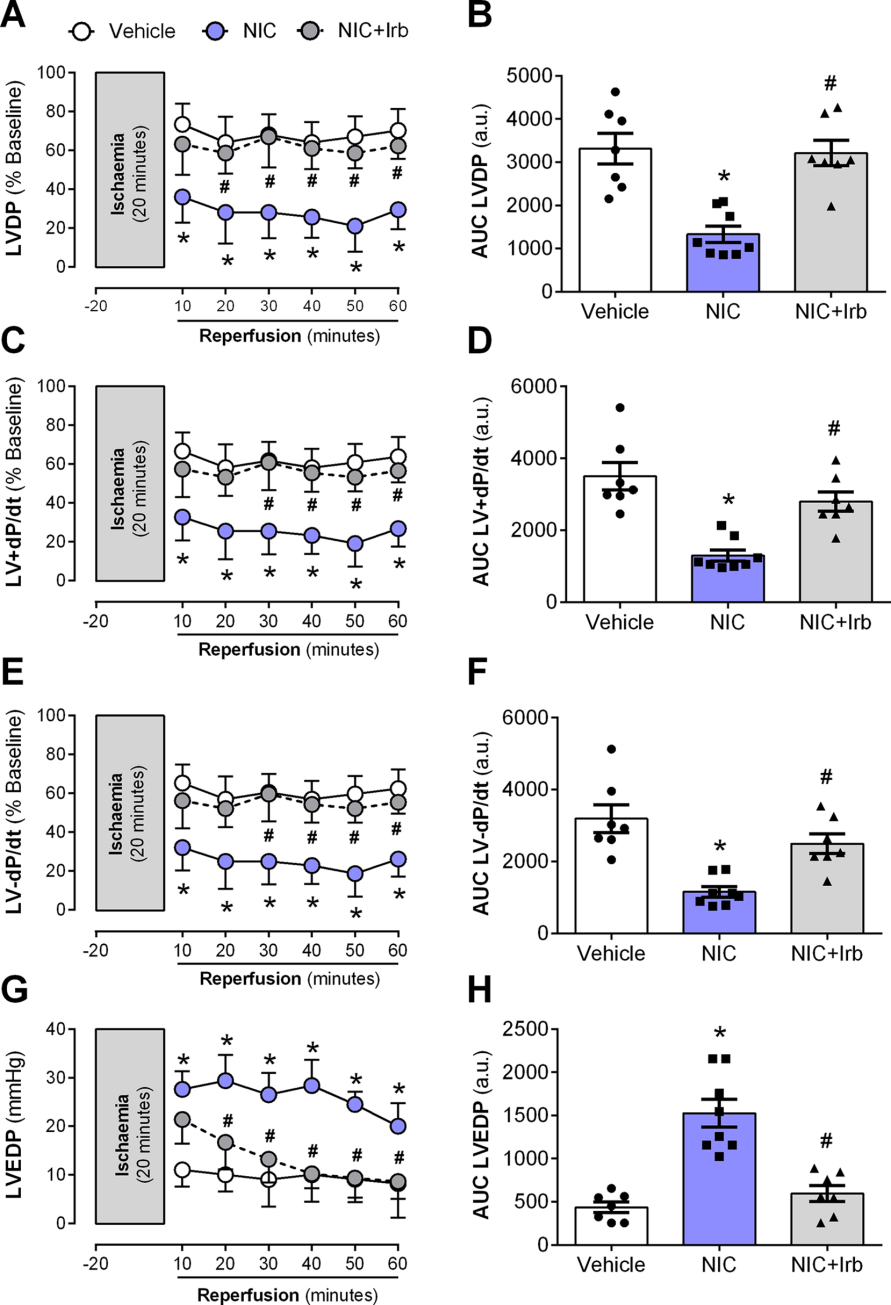
AT1 receptor antagonism abolishes nicotine-aggravation of post-ischemic left ventricle dysfunction. Time-course and area-under-curve (AUC) analysis for changes in **(A**–**B)** left ventricular developed pressure, **(C**–**D)** LV+dP/dt, **(E**–**F)** LV-dP/dt, and **(G**–**H)** left ventricular end-diastolic pressure in Langendorff-perfused rat hearts isolated from nicotine and irbesartan-administered rats during 20 min of ischemia and 60 min of reperfusion. All values are given as mean ± SEM for n = 7 –8/group; *P < 0.05 for control *vs.* nicotine (NIC) group; and ^#^ *P* < 0.05 for NIC *vs.* NIC+Irb group using two-way ANOVA with Tukey *post-hoc* test.

Langendorff-perfused hearts isolated from nicotine-administered rats exhibited severe LV dysfunction following 20 min of ischemia and 60 min of reperfusion. Nicotine significantly impaired recovery of LVDP evident from 10 min after the onset of reperfusion (*P* < 0.05; **Figure 6A**). In addition, the area-under-the-curve (AUC) for LVDP recovery was significantly diminished in nicotine-administered rats compared to the vehicle controls (*P* < 0.05; **Figure 6B**). A similar trend was observed for recovery of both LV+dP/dt and LV-dP/dt (all *P* < 0.05; **Figures 6C–F**). LVEDP was significantly elevated in nicotine-administered rats compared to vehicle controls throughout the full 60 min of reperfusion, indicative of impaired LV relaxation (*P* < 0.05; **Figure 6G**). Consistent with this, the AUC for LVEDP was also elevated in the nicotine-administered experimental group compared to vehicle controls (*P* < 0.05; **Figure 6H**). In contrast, irbesartan significantly improved post-ischemic recovery of LV function, as shown across LVDP, LV+dP/dt, and LV-dP/dt in both time course and AUC analysis (all *P* < 0.05; **Figures 6A–F**). Irbesartan also prevented the nicotine-induced elevation in LVEDP in reperfused rat hearts (*P* < 0.05; **Figures 6G–H**).

### Impact of AT1 Antagonism on Nicotine-Aggravation of Myocardial Injury

**Figure 7:**
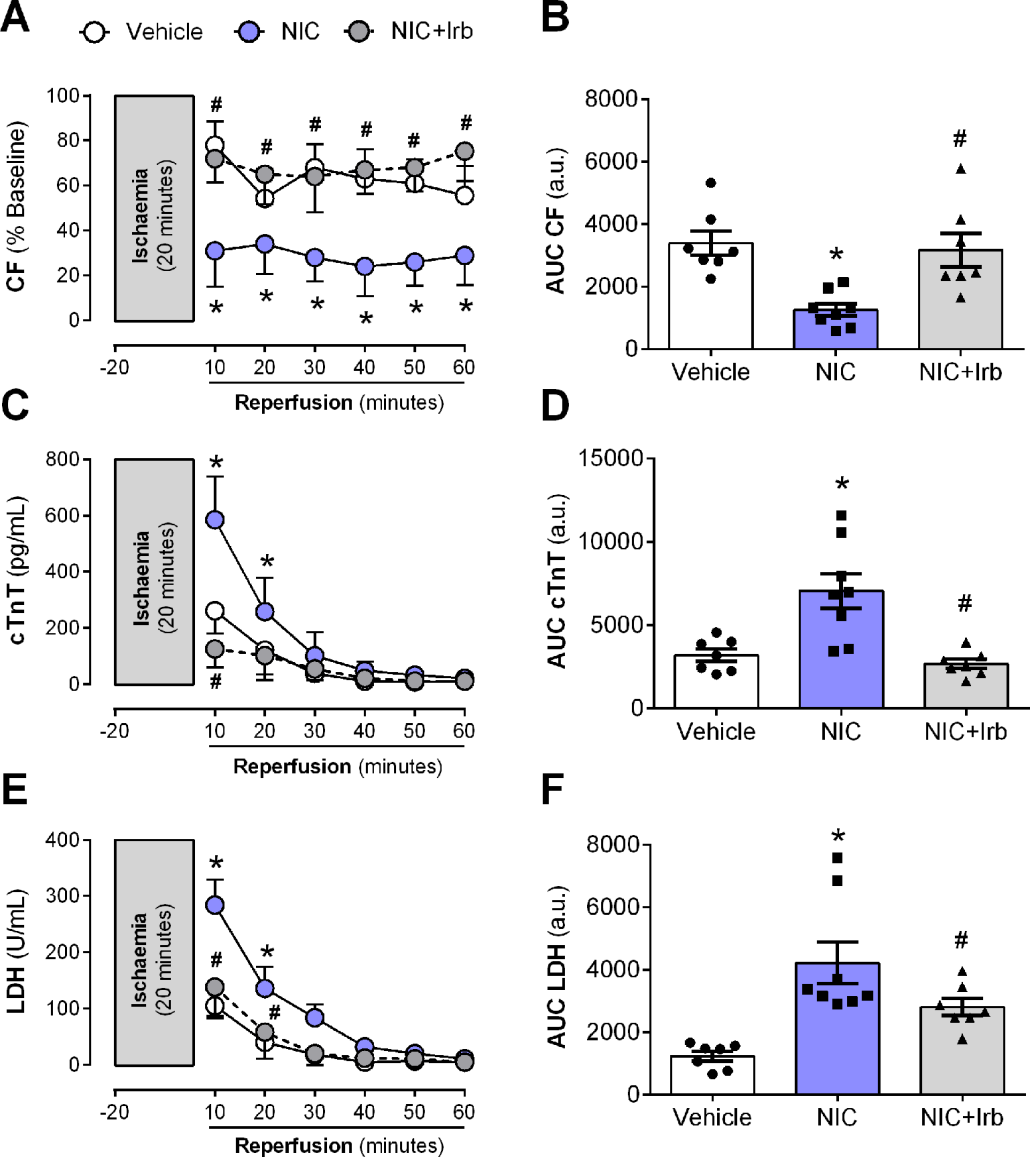
AT1 receptor antagonism prevents nicotine-induced post-ischemic impairment in coronary function and cardiomyocyte injury. Time-course and area-under-curve (AUC) analysis for changes in **(A**–**B)** coronary flow, **(C**–**D)** cardiac troponin T (cTnT) release, and **(E**–**F)** lactate dehydrogenase release from Langendorff-perfused rat hearts isolated from nicotine and irbesartan-administered rats during 20 min of ischemia and 60 min of reperfusion. All values are given as mean ± SEM for n = 7 –8/group; *P < 0.05 for control *vs.* nicotine (NIC) group; and ^#^ *P* < 0.05 for NIC *vs.* NIC+Irb group using two-way ANOVA with Tukey *post-hoc* test.

The nicotine-induced impairment in CF was further exacerbated following I/R, as shown across both recovery of flow and AUC analysis (both *P* < 0.05; **Figures 7A, B**). This was accompanied by enhanced efflux of cardiac injury markers, cTnT and LDH from the onset of reperfusion (all *P* < 0.05; **Figures 7C–F**). Irbesartan not only prevented the nicotine-induced aggravation of coronary dysfunction bur it also lowered the release of cTnT and LDH from Langendorff-perfused rat hearts (all *P* < 0.05; **Figure 7**).

## Discussion

Nicotine has been suggested to play a prominent role in the development of cardiovascular diseases such as hypertension and atrial fibrillation (Goette, 2009; Harwani et al., 2016; Shao et al., 2017; Zuo et al., 2017). Evidence from animal and *in vitro* studies, including our own has suggested that nicotine may directly impact structure and function of myocardium, rendering it more vulnerable to acute I/R injury (Hu et al., 2011; Li et al., 2016; Ramalingam et al., 2016). Although oxidative stress has been implicated as a key mechanism contributing to the detrimental effects of nicotine (Gumustekin et al., 2010; Zainalabidin et al., 2016); precise mechanisms underlying nicotine-induced cardiac dysfunction remain unclear. In this study, we have demonstrated for the first time that AT1 receptor, a positive regulator of ROS production; is key player in nicotine-induced cardiac dysfunction. Our major findings were that: i) prolonged nicotine administration for 28 days up-regulated deleterious cardiac ACE-ANG II-AT1 axis in rats, and ii) selective antagonism of AT1 receptors using irbesartan effectively attenuated nicotine-induced cardiac dysfunction, and abolished nicotine-aggravation of myocardial susceptibility to I/R injury in these rats. Irbesartan may have protected against nicotine-induced cardiac dysfunction, in part *via* reduction of blood pressure, cardiac hypertrophy, fibrosis, oxidative stress, and inflammation.

Renin-angiotensin system (RAS) is a significant contributor to development of cardiovascular diseases (Wu et al., 2018). Although several studies have shown that nicotine affects expression of ACE and ACE2 in endothelial cells *in vitro* as well as other organs *in vivo* (Saijonmaa et al., 2005; Ljungberg and Persson, 2008; Oakes et al., 2018), there remains limited data available on the impact of prolonged nicotine administration on cardiac RAS expression *in vivo*. In this study, we showed that nicotine administration for 28 days significantly up-regulated cardiac level of ANG II, gene expression of ACE, ACE : ACE2 messenger RNA (RNA) ratio, as well as AT : AT2 mRNA. Nicotine also markedly reduced cardiac gene expression of ACE2 in these rats without affecting the AT2 expression. Overall, this suggested that prolonged nicotine up-regulated the deleterious ANG II-ACE-AT1 axis at gene expression level which has been associated with various cardiovascular diseases and phenotypes such as cardiac hypertrophy, fibrosis, and oxidative stress (Pernomian et al., 2014; Wang et al., 2016). In contrast, irbesartan administration significantly down-regulated AT1 gene expression together with mRNA ratios of ACE : ACE2 as well as AT1:AT2 while showing a tendency to lower ANG levels in plasma and heart. This suggested that irbesartan co-administration blunted nicotine-induced upregulation the ANG II-ACE-AT1 axis in the heart, at least at gene expression level. Future studies should determine whether these observations are seen at protein level as well.

Nicotine-induced cardiac dysfunction is often associated with structural changes such as cardiac hypertrophy and fibrosis (Goette et al., 2007; Hu et al., 2011). Cardiac hypertrophy is initially an adaptive process that allows cardiac muscles to grow in mass to withstand increases in pressure or volume overload in settings such as high blood pressure. Such growth in muscle mass, however may transition into pathological hypertrophy if pressure or volume overload remain unrelieved (Tham et al., 2015). Pathological cardiac hypertrophy is clinically significant, as it is an independent risk factor for both heart failure and sudden cardiac death (Stevens et al., 2013; Okwuosa et al., 2015). Within myocardium, pathological hypertrophy manifests four distinct characteristics, which are i) increased cardiomyocyte size, ii) re-activation of fetal genes such as ANP and BNP, iii) presence of fibrosis, as well as iv) depressed cardiac function (Bernardo et al., 2010). In this study, nicotine administration for 28 days significantly increased indices of heart mass (HW : TL and LV : TL), cardiomyocyte size (cardiomyocyte CSA), as well as gene expression of ANP and BNP, supporting that nicotine induces pathological cardiac hypertrophy. Collagen deposition was also enhanced in nicotine-administered rat hearts. While increased SBP and upregulated ACE-ANGII-AT1 axis may have contributed predominantly to nicotine-induced cardiac hypertrophy observed, recent *in vitro* studies have also suggested that nicotine may directly increase cardiomyocyte size through calcineurin/NFAT signaling and ROS production (Li et al., 2016). Increased mitochondrial ROS and oxidative stress production, as noted in this study could also be responsible cardiac hypertrophy evident in nicotine-administered rats (Dai et al., 2011).

In addition to its role in development of pathological cardiac hypertrophy, an extensive amount of studies had shown that fibrosis *per se* is associated with heart failure (Schelbert et al., 2017). Increased collagen density within myocardium not only increases stiffness of LV muscles; but also reduces diastolic compliance necessary for ventricular blood filling (Røe et al., 2017). Severe fibrosis evident from increased collagen deposition in nicotine-administered rats, may therefore account for impaired cardiac function observed in this study. Several *in vitro* studies have shown that nicotine directly increases fibroblast proliferation and collagen deposition *via* modulation of pro-fibrotic cytokines such as TGFβ, microRNAs, and matrix metalloproteinases (Shan et al., 2009; Khoi et al., 2013; Ebrahimpour et al., 2018). Indeed, TGFβ was widely implicated as a key signaling molecule responsible for nicotine-induced fibrosis in various organs and cells (Rezonzew et al., 2012; Ateyya et al., 2017). TGFβ is a crucial signaling molecule necessary for regulation of fibroblast proliferation, migration, and differentiation. Evidence from cross-sectional studies had shown that level of TGFβ independently predicts incidence of coronary artery disease and heart failure in humans, making it an excellent biomarker for fibrosis (Chen et al., 2014). We have thus shown here that nicotine increases LV gene expression of TGFβ1 and this may be responsible for nicotine-induced cardiac fibrosis. We have also observed that nicotine concomitantly increased gene expression of fibronectin (FN1 gene) in rat hearts after 28 days. Unlike TGFβ1, it is unclear whether increased FN1 expression is a mechanism or consequence of fibrosis; however, it has been shown in transgenic animals that systemic FN1 knockout prevents pressure-overload induced cardiac hypertrophy and fibrosis (Konstandin et al., 2013). Mice lacking FN1 extra domain A also exhibited reduced fibrosis and ventricular dilatation following acute MI (Arslan et al., 2011). FN1 therefore may also be necessary for nicotine-induced cardiac fibrosis similar to TGFβ1; however, future studies are warranted to define precise role of FN1 in nicotine-induced cardiac fibrosis. Regardless, this study also highlighted that nicotine-induced cardiac remodeling may also underlie aggravation of myocardial I/R injury after prolonged nicotine administration Evidence from isolated animal heart studies and human trials showed that remodeled hearts are more vulnerable to I/R injury due to increased oxygen demand, oxidative stress, muscle stiffness, and poor diastolic compliance (Ma et al., 2017).

In this study, AT1 receptor antagonism using irbesartan successfully prevented nicotine-induced cardiac hypertrophy and fibrosis. Irbesartan significantly abolished nicotine-induced increases in cardiomyocyte size, collagen deposition, as well as LV gene expression of ANP, BNP, and FN1. Irbesartan also tended to reduce LV gene expression of TGF-β1 as well as indices of heart mass (HW : TL and LV : TL). This altogether suggested that activation of AT1 receptors may underlie development of cardiac hypertrophy and fibrosis in nicotine-administered rats. Nonetheless, it should be noted that blood pressure lowering effect of irbesartan may have also prevented nicotine-induced cardiac hypertrophy and fibrosis (Watanabe et al., 2015), considering hypertension itself is a key driving force for adverse cardiac remodeling (González et al., 2018). For these reasons, we can only speculate that a combination of both AT1 receptor antagonism and blood pressure lowering could have mediated the prevention of nicotine-induced cardiac remodeling in irbesartan-administered animals in this study. Future studies that could potentially identify whether AT1 receptor antagonists prevents nicotine-induced cardiac remodeling in absence of hypertension are therefore warranted. Nonetheless, it is possible that irbesartan-mediated prevention of cardiac remodeling may have contributed in part to the reduced susceptibility to I/R injury in hearts isolated from NIC+Irb group as compared to those from NIC alone group.

Oxidative stress has been implicated in a majority of studies, as a key mechanism contributing to nicotine-induced cardiac dysfunction (Zhou et al., 2010; Hu et al., 2011; Ramalingam et al., 2016). In rat models of prolonged nicotine administration, nicotine administration increased levels of cardiac lipid peroxidation and protein oxidation within 21 to 28 days (Gumustekin et al., 2010; Zainalabidin et al., 2016). Consistent with these previous studies, we have shown that nicotine administration induced oxidative stress in rat hearts, as shown across GSH : GSSG ratio, 3-nitrotyrosine level, increased NOX2 gene expression, as well as mitochondrial ROS generation in this study. NADPH oxidase and mitochondria are both major sources of ROS in the heart (Huynh et al., 2014). NOX2 subunit of NADPH oxidase, in particular is strongly linked to adverse cardiac remodeling whereby NOX2 deficiency alone completely prevented oxidative stress and LV dysfunction in animal models of acute MI and pressure overload-induced hypertrophy (Looi et al., 2008; Parajuli et al., 2014). Mitochondrial ROS is also similarly important for pathogenesis of adverse cardiac remodeling and dysfunction. Studies have shown that mitochondria-targeted antioxidants ameliorated cardiac remodeling and functional defects in animal models of pressure-overload induced hypertrophy and diabetes (Ni et al., 2016; Goh et al., 2019). Nicotine administration for 28 days also significantly reduced activity of SOD2 without affecting its gene expression in this study. SOD2 is an isoform of SOD2 enzyme that is responsible for ROS scavenging in mitochondria (Kang et al., 2015). Altogether, these markers of oxidative stress highlights that ROS-driven oxidative stress may underlie pathogenesis of cardiac hypertrophy, fibrosis, and LV dysfunction. Increased ROS production may also reduce nitric oxide bioavailability, rendering to nicotine-induced impairment in coronary vasodilation observed in isolated heart experiments.

Compared to the untreated nicotine group, irbesartan significantly blunted myocardial oxidative stress in this study as shown by preserved GSH : GSSG ratio and reduction across 3-nitrotyrosine level, NOX2 gene expression, and mitochondrial ROS production. Irbesartan also tended to preserve SOD2 activity in these rats. The mechanisms by which irbesartan blunted oxidative stress was unexplored in this study, however previous studies have abundantly shown that ANG II is a direct regulator of NADPH oxidase and mitochondrial ROS production *via* activation of AT1 receptors (Dikalov and Nazarewicz, 2013; Murdoch et al., 2014). Therefore, AT1 blockade may have directly reduced ROS production in nicotine-administered rats. These observations suggested that AT1-mediated oxidative stress also most likely contributed in part to the nicotine-induced cardiac remodeling and dysfunction. Irbesartan-mediated reduction in oxidative stress may also have conferred protection against I/R injury in this study, considering a past study has reported that irbesartan pre-treatment attenuated oxidative stress and injury in HL-1 cells subjected to hypoxia *in vitro* (Boccellino et al., 2018).

Unresolved inflammation is also documented as a key player in pathogenesis of cardiac hypertrophy, fibrosis, and eventual LV dysfunction (Qin et al., 2019). Cardiac expression of pro-inflammatory cytokines such as TNFα and IL6 often increases in settings of cardiac distress such as diabetic cardiomyopathy and acute MI (Tate et al., 2017; Ali et al., 2018), and this is necessary for tissue repair and healing. Unregulated, persistent inflammation is however detrimental to the myocardium, as chronic activation of TNFα and IL6 signaling causes extensive loss of cardiomyocytes, severe fibrosis, and decompensated LV dysfunction (Haudek et al., 2007; Mele´ndez et al., 2010). Several lines of evidence have now reported that nicotine has pro-inflammatory actions in the cardiovascular system. In rats, nicotine administration for 21 days caused significant up-regulation of TNFα and myeloperoxidase accumulation in heart tissues (Demiralay et al., 2007). Nicotine also increased gene expression of TNFα and inducible nitric oxide synthase in mouse peritoneal macrophages and RAW264.6 macrophages *in vitro* (Lau et al., 2006). Consistent with these previous observations, nicotine induced significant increases in cardiac TNFα and IL6 gene expression in this study, indicative of unresolved chronic inflammation. Blockade of AT1 receptor using irbesartan was able to completely attenuate nicotine-induced increases in TNFα and IL6 gene expression. Our findings were consistent with past studies which have reported anti-inflammatory actions of irbesartan in settings of post-MI cardiac remodeling and hypertension (Taguchi et al., 2013; Watanabe et al., 2015).

We have also shown for the first time that nicotine up-regulated cardiac gene expression of ANXA1 and its receptor, FPR2 following 28 days of administration. ANXA1 is an endogenous glucocorticoid-regulated peptide, whose expression is necessary for resolution of inflammation (Qin et al., 2012; Qin et al., 2019). Activation of ANXA1/FPR2 signaling is widely shown to limit inflammation *via* accelerated neutrophil clearance and production of anti-inflammatory cytokines such as IL10 (Ferlazzo et al., 2003; Dalli et al., 2012). In this study, cardiac IL10 gene expression was not increased in nicotine-administered rats, in spite of up-regulated ANXA1/FPR pathway. ANX-A1 up-regulation is likely a compensatory response toward inflammatory stimulus in nicotine-administered rats; lack of IL10 however may have prevented resolution of inflammation in these rats. Deficiency of endogenous IL10 alone has been shown to worsen progression of inflammation, fibrosis, and LV dysfunction in mouse model pressure overload-induced hypertrophy (Verma et al., 2012). In contrast to the nicotine group, irbesartan-administered rats had significantly elevated IL10 expression, in addition to up-regulated ANXA1/FPR2 as compared to the controls. Up-regulated IL10 might have contributed partly to the beneficial effects of irbesartan seen in this study. The mechanisms by which nicotine or irbesartan affected ANXA1/FPR2 pathway was unexplored in this study, however future studies are highly warranted given that targeting this pathway causes cardioprotection in various settings.

### Study Limitation

One of the major limitations of this study is the lack of *in vivo* cardiac function data using echocardiography. We acknowledge that isolated Langendorff perfused heart does not entirely replicate the *in vivo* setting and function analysis; where the heart works against preload and afterload, as well as fail to take into account influence from circulating inflammatory cells, hormones, and chemokines. Nonetheless, this approach using Langendorff perfusion allows examination of changes in intrinsic cardiac contractile function in a whole heart that is beating spontaneously. Echocardiography is the most commonly used method for routine detection of cardiac dysfunction in clinical settings, therefore, future studies should examine the impact of nicotine administration, alone or in combination with AT1 receptor antagonist on echocardiographic measures of cardiac function *in vivo*.

## Conclusion

This is the first study to demonstrate that AT1-receptor activation is necessary for nicotine-induced cardiac dysfunction and associated aggravation of myocardial I/R injury. We showed that AT1 receptor antagonism using irbesartan prevented nicotine-induced cardiac dysfunction *via* reduction of myocardial hypertrophy, fibrosis, oxidative stress, and inflammation. Improvement in endogenous antioxidant status, cardiac structure, and resolved inflammation as well as reduced blood pressure may also have prevented nicotine aggravation of myocardial I/R injury. Additional studies to explore the impact of RAS antagonists on nicotine-induced cardiac dysfunction may provide stronger evidence for use of these drugs to manage cardiovascular complications seen in tobacco users.

## Data Availability Statement

The datasets generated for this study are available on request to the corresponding author.

## Ethics Statement

The animal study was reviewed and approved by the Universiti Kebangsaan Malaysia Animal Ethics Committee, Kuala Lumpur, Malaysia.

## Author Contributions

Study design: AR, RR, and SZ. Study conduct: AR and SZ. Data collection and analysis: AR. Data interpretation: All authors. Drafting manuscript: AR. Revising manuscript content: All authors. Approving final version of manuscript: All authors. AR takes responsibility for the integrity of the data analysis.

## Funding

This work was supported by the Fundamental Research Grant Scheme from the Ministry of Higher Education, Malaysia (FRGS/2/2014/SG03/UKM/02/3).

## Conflict of Interest

The authors declare that the research was conducted in the absence of any commercial or financial relationships that could be construed as a potential conflict of interest.
