# Supplementary material for: Angiotensin II Type I Receptor Antagonism Attenuates Nicotine-Induced Cardiac Remodeling, Dysfunction, and Aggravation of Myocardial Ischemia-Reperfusion Injury in Rats
Source: Front Pharmacol. 2019 Dec 12;10:1493. doi: 10.3389/fphar.2019.01493 (PMC6920178; doi:10.3389/fphar.2019.01493)
Supplement: Supplementary file 1 [file DataSheet_1.docx]

Online Supplement

**Angiotensin II Type I Receptor Antagonism Attenuates Nicotine-Induced Cardiac Remodelling, Dysfunction and Aggravation of Myocardial Ischaemia-Reperfusion Injury in Rats**

**Anand Ramalingam^1^, Siti Balkis Budin^1^, Norsyahida Mohd. Fauzi^2^, Rebecca H. Ritchie^3†^,**

**Satirah Zainalabidin^1†*^**

1. Programme of Biomedical Science, Centre for Allied and Health Sciences, Faculty of Health Sciences, Universiti Kebangsaan Malaysia, Kuala Lumpur 50300, Malaysia
2. Drug and Herbal Research Centre, Faculty of Pharmacy, Universiti Kebangsaan Malaysia, Jalan Raja Muda Abdul Aziz, Kuala Lumpur 50300, Malaysia
3. Heart Failure Pharmacology, Baker Heart and Diabetes Institute, Melbourne, Victoria 3004, Australia

† Joint Senior Authors

***Correspondence:**

Satirah Zainalabidin, Ph.D.

**Email:** satirah@ukm.edu.my

**Keywords:** **Cardiac fibrosis, Hypertension, Inflammation, Irbesartan; Oxidative stress**

**Abstract**

Increased exposure to nicotine contributes to the development of cardiac dysfunction by promoting oxidative stress, fibrosis and inflammation. These deleterious events altogether render cardiac myocytes more susceptible to acute cardiac insults such as ischaemia-reperfusion (I/R) injury. This study sought to elucidate the role of angiotensin II type I (AT1) receptors in cardiac injury resulting from prolonged nicotine administration in a rat model. Male Sprague-Dawley rats were given nicotine (0.6 mg/kg ip) for 28 days to induce cardiac dysfunction, alone or in combination with the AT1 receptor antagonist, irbesartan (10 mg/kg, po). Vehicle-treated rats were used as controls. Rat hearts isolated from each experimental group at study endpoint were examined for changes in function, histology, gene expression and susceptibility against acute I/R injury determined ex vivo. Rats administered nicotine alone exhibited significantly increased cardiac expression of angiotensin II and angiotensin-converting enzyme (ACE) in addition to elevated systolic blood pressure (SBP) and heart rate. Furthermore, nicotine administration markedly reduced left ventricular (LV) performance with concomitant increases in myocardial oxidative stress, fibrosis and inflammation. Concomitant treatment with irbesartan attenuated these effects, lowering blood pressure, heart rate, oxidative stress and expression of fibrotic and inflammatory genes. Importantly, the irbesartan-treated group also manifested reduced susceptibility to I/R injury ex vivo. These findings suggest that AT1 receptors play an important role in nicotine-induced cardiac dysfunction, and pharmacological approaches targeting cardiac AT1 receptors may thus benefit patients with sustained exposure to nicotine.

**Supplementary Figure**

**A**


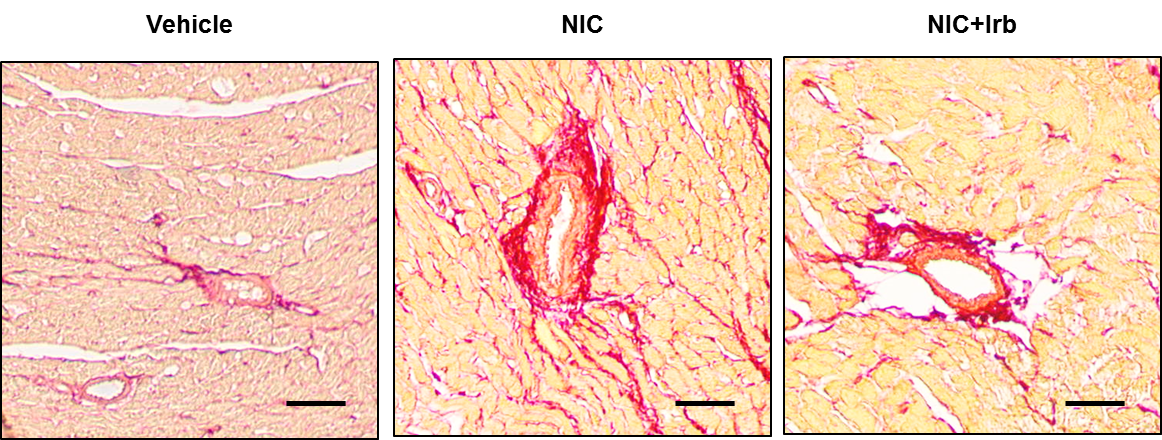


**B**





**Figure S1.** Perivascular fibrosis in rat myocardium after 28 days of nicotine and irbesartan administration. (A) Representative images of LV sections-stained with picrosirius red from each group and (B) semi-quantitative analysis of perivascular collagen density. All values are given as mean ± SEM for n=6-7/group; NS, no significant difference using one-way ANOVA with Tukey post-hoc test. Neither nicotine nor irbesartan had significant effect on perivascular fibrosis in this study; although there was a trend for increased perivascular collagen density in the nicotine alone group.
